# Reconsidering the Sedentary Behaviour Paradigm

**DOI:** 10.1371/journal.pone.0086403

**Published:** 2014-01-15

**Authors:** Carol Maher, Tim Olds, Emily Mire, Peter T. Katzmarzyk

**Affiliations:** 1 Health and Use of Time Group, School of Health Sciences, University of South Australia, Adelaide, South Australia, Australia; 2 Division of Population Science, Pennington Biomedical Research Centre, Baton Rouge, Louisiana, United States of America; Pennington Biomed Research Center, United States of America

## Abstract

**Aims:**

Recent literature has posed sedentary behaviour as an independent entity to physical inactivity. This study investigated whether associations between sedentary behaviour and cardio-metabolic biomarkers remain when analyses are adjusted for total physical activity.

**Methods:**

Cross-sectional analyses were undertaken on 4,618 adults from the 2003/04 and 2005/06 U.S. National Health and Nutrition Examination Survey. Minutes of sedentary behaviour and moderate-to-vigorous physical activity (MVPA), and total physical activity (total daily accelerometer counts minus counts accrued during sedentary minutes) were determined from accelerometry. Associations between sedentary behaviour and cardio-metabolic biomarkers were examined using linear regression.

**Results:**

Results showed that sedentary behaviour was detrimentally associated with 8/11 cardio-metabolic biomarkers when adjusted for MVPA. However, when adjusted for total physical activity, the associations effectively disappeared, except for C-reactive protein, which showed a very small, favourable association (β = −0.06) and triglycerides, which showed a very small, detrimental association (β = 0.04). Standardised betas suggested that total physical activity was consistently, favourably associated with cardio-metabolic biomarkers (9/11 biomarkers, standardized β = 0.08–0.30) while sedentary behaviour was detrimentally associated with just 1 biomarker (standardized β = 0.12).

**Conclusion:**

There is virtually no association between sedentary behaviour and cardio-metabolic biomarkers once analyses are adjusted for total physical activity. This suggests that sedentary behaviour may not have health effects independent of physical activity.

## Introduction

In recent years, a substantial literature has identified sedentary behaviour as an independent risk factor for a wide range of health outcomes. [1], [2], [3], [4], [5] It has been postulated that there is something about sedentary behaviour in itself — i.e. other than the fact that it displaces other types of physical activity — which causes cardio-metabolic deficits. For example, postural stasis, triggering a chain of unhealthy molecular sequelae, may be a critical mechanism. [6]

The persistent relationship between sedentary behaviour and cardio-metabolic deficits, even when adjusted for physical activity, has been cited as evidence of sedentary behaviour's unique health effects. [1], [2] However, intriguingly, epidemiological studies on which these assertions are based have adjusted only for limited measures of physical activity, such as duration of moderate-to-vigorous physical activity (MVPA), [3] or even subcomponents of MVPA, such as leisure time MVPA. [4], [5] This approach has a number of limitations. It treats MVPA as a homogeneous entity, whereas moderate physical activity and vigorous physical activity are recognized to have distinct physiologic effects. [7] Furthermore, light physical activity (eliciting between 1.5 and 3 METs) has been overlooked, and there is a growing body of literature that light physical activity has health benefits. [8], [9] This is important, particularly when considering that light physical activity constitutes a relatively large part of the day for most adults when compared to MVPA. [10]

In the largest epidemiological study to date based upon the National Health and Nutrition Examination Survey (NHANES) dataset, Healy and colleagues stated that their analyses could not be controlled for light physical activity because light physical activity and sedentary behaviour were almost perfectly inversely associated (Spearman's rho = −0.98), and thus would have caused collinearity in the regression models. [11] However, if these variables are virtually the inverse of one another, then the detrimental associations identified between sedentary time and various cardio-metabolic biomarkers might, in fact, be markers of favourable associations between light physical activity and the biomarkers.

To truly ascertain whether sedentary behaviour and cardio-metabolic markers are related independent of physical activity, studies are needed which control for total physical activity (i.e. light, moderate and vigorous activity). If associations between sedentarism and health outcomes disappear, or are largely attenuated, when corrected for total physical activity, then displacement would appear to be the most likely mechanism.

Accounting for all types of physical activity (light, moderate and vigorous) is not possible if analyses are based on duration derivatives of accelerometry (e.g. minutes of sedentary time, minutes of light physical activity and so on); collinearity will necessarily occur, given that time in all bands will add up to 24 hours per day (adjusted for wear time). However, counts-based derivatives of accelerometry (e.g. total daily accelerometer counts) might alleviate the issue of collinearity, since total daily accerelometer counts do not have a fixed upper limit, and will vary from person to person, and day to day.

This study aimed to investigate the associations between sedentary behaviour and cardio-metabolic biomarkers when corrected for total physical activity (including light, moderate and vigorous physical activity). In addition, we aimed to compare the associations between sedentary behaviour and cardio-metabolic biomarkers when adjusting for total physical activity to those identified when adjusting for a more conventional measure of physical activity, namely MVPA minutes. Analyses were undertaken using a large, representative sample of US adults from the 2003/4 and 2005/6 waves of NHANES.

## Materials and Methods

### Ethics Statement

The survey complies with the Declaration of Helsinki, The National Center for Health Statistics Ethics Review Board approved the protocols, and participants provided written informed consent. See http://www.cdc.gov/nchs/nhanes.htm for full methodological details.

### Design and participants

NHANES is a large cross-sectional survey, representative of the US civilian non-institutionalized population. Participants complete in-home questionnaires and a physical examination at a Mobile Examination Center. Participants were included in this study if they were aged ≥20 years, participated in the NHANES 2003/4 or 2005/6 waves, and had valid accelerometer data. Pregnant women and those taking insulin were excluded, as were those with missing cardio-metabolic outcomes and continuous covariates.

### Cardio-metabolic variables

Waist circumference, resting systolic and diastolic blood pressure, non-fasting HDL-cholesterol and non-fasting C-reactive protein concentration were measured during the physical examination. In addition, approximately half the NHANES sample provided a fasting blood sample, from which fasting triglycerides, fasting plasma glucose and fasting insulin was determined. Samples were excluded from analyses if participants reported that they fasted for less than 8.5 hours. In 2005/06 only, the fasting subsample also underwent a 2 h oral glucose tolerance test (OGTT) to produce 2 h plasma glucose values.

To account for differences in methods for measuring fasting glucose and insulin between 2003/04 and 2005/06, correction equations released by NHANES were applied (downloaded from http://www.cdc.gov/nchs/nhanes/nhanes2005-2006/GLU_D.htm). However, very low 2005/06 insulin values became improbably low following correction (in some cases, negative). In these instances, the 2005/06 data were assigned the lowest insulin value measured in the 2003/04 cohort (4.931 pmol/L). The corrected values were then used to calculate Homeostatic Model Assessment beta-cell function (HOMA-%B) and insulin sensitivity (HOMA-%S), using the University of Oxford (2004) HOMA calculator (http://www.dtu.ox.ac.uk/homacalculator/download.php).

### Accelerometry

The 2003/4 and 2005/6 NHANES protocol asked ambulatory participants to wear an Actigraph 7164 accelerometer (Actigraph, LLC, Fort Walton Beach, FLA) on their right hip during waking hours (except water activities) for seven days (see [12] for the full accelerometry protocol).

An automated program available from the National Cancer Institute website was used to carry out quality control procedures, derive wear time, and summarize minute-by-minute data (http://riskfactor.cancer.gov/tools/nhanes_pam/create.html). Specifically, non-wear time was defined on the basis of 60 consecutive minutes of 0 counts per minute (cpm), with allowance for up to 2 minutes of <100 cpm. The data were censored so that minutes with ≥20,000 cpm were considered invalid, with the average of valid intensity counts immediately before and after such invalid minute(s), imputed to replace the invalid minute(s). Days with at least 10 hours of wear time were considered valid. Since we were interested in behaviour patterns, only participants with at least four valid days, including at least one weekend day, were included in analyses.

For each participant, MVPA time was calculated as the mean daily minutes ≥2020 cpm from all valid days. [13] Mean daily sedentary time was calculated as the mean daily minutes <100 cpm, minus non-wear time. [14] Total physical activity was calculated as the total daily accelerometer counts minus counts accrued during sedentary minutes, averaged across valid days.

### Socio-demographic covariates

Age in years and sex were recorded during the screening interview. Socio-demographic variables (ethnicity, household income and highest educational attainment) recorded in the household questionnaires, were collapsed into categories (Table 1). In order to retain the maximum number of participants in analyses, missing categorical data were recoded into “missing” categories. Poverty to income ratio was provided in the NHANES datasets, and was calculated by the Centers for Disease Control and Prevention by dividing family income by poverty guidelines, specific to family size, year and state. [15]

**Table 1:** Population Weighted Descriptive Characteristics of Adults in the 2003/04 and 2005/06 U.S. National Health and Nutrition Examination Survey.

Socio-demographic		Women	Men
		2216 (48.0%)	2402
Age (%)	20–39 years	28.6	33.2
	40–59 years	42.0	42.6
	60+ years	29.4	24.1
Ethnicity (%)	Mexican American	6.4	8.4
	Other Hispanic	3.4	2.5
	Non-Hispanic White	75.9	76.6
	Non-Hispanic Black	9.0	8.1
	Other	5.3	4.5
Educational attainment (%)	Less than 9^th^ grade	5.2	6.5
	9^th^-12^th^ grade with no high school diploma	9.0	9.3
	High school diploma or equivalent	25.4	24.5
	Some post high school	33.7	31.1
	College graduate or higher	26.7	28.4
	Missing^a^	0.02	0.04
Household income (%)	$ 0 to $ 4,999	0.7	1.1
	$ 5,000 to $ 9,999	3.2	1.9
	$10,000 to $14,999	5.3	3.7
	$15,000 to $19,999	5.5	4.6
	$20,000 to $24,999	6.7	5.2
	$25,000 to $34,999	12.2	11.3
	$35,000 to $44,999	9.8	10.0
	$45,000 to $54,999	9.8	11.1
	$55,000 to $64,999	7.9	7.9
	$65,000 to $74,999	5.9	7.0
	$75,000 and Over	27.7	32.2
	Over $20,000	1.2	1.0
	Under $20,000	0.2	0.2
	Missing^a^	3.7	2.8
BMI (kg/m^2^); mean (SE)		28.0 (0.2)	28.2 (0.1)
Overweight (%)		30.5	41.6
Obese (%)		31.3	31.1
**Behaviours**			
Daily MVPA minutes; mean (SE)		17.1 (0.6)	29.7 (0.7)
Daily total accelerometer activity counts mean (SE)		244,314 (3390.9)	305,505 (3467.6)
Daily sedentary minutes mean (SE)		484.5 (1.9)	490.8 (3.2)
Sedentary quartiles (minutes) mean (SE)	1	353.9 (2.1)	339.6 (1.9)
	2	453.1 (1.0)	450.6 (1.3)
	3	524.5 (1.2)	527.4 (0.9)
	4	630.1 (4.1)	642.9 (3.0)
Daily accelerometer wear time mean (SE)		850.0 (3.2)	870.2 (2.9)
Daily total energy intake (kcal) mean (SE)		1794.0 (16.0)	2561.8 (18.6)
Saturated fat as % of total energy mean (SE)		11.2 (0.1)	11.2 (0.1)
Alcohol intake (%)	None	71.5	57.1
	Light (men <28 g/d; women <14 g/d),	14.9	23.7
	Moderate (men 28 – <56 g/d; women 14 – <28 g/d)	7.3	11.4
	Heavy (men ≥56 g/d; women ≥28 g/d)	6.2	7.8
Smoking status (%)	Non (<10 ng/dL)	81.9	69.1
	Light (10 – <100 ng/dL)	3.4	6.8
	Moderate (100 – <300 ng/dL)	10.1	15.1
	Heavy (≥300 ng/dL)	4.6	8.9
	Missing^a^	0.05	0.1

Abbreviations: BMI =  body mass index; MVPA =  moderate to vigorous physical activity; SE =  standard error.

^a^ Missing category created to retain participants with missing data in analyses.

### Medical history covariates

Medical and family medical history data were collected using the household questionnaires. Categorical variables were created for family history of stroke/hypertension, angina and diabetes (“no”, “yes” or “missing”) and medical history of cancer, cardiovascular disease (“no”, “yes” or “missing”) and diabetes (“no”, “yes”, “pre-diabetes” or “missing”). Current medication use was recorded, and coded using the Lexicon Plus (Cerner Multum Inc.) database, to form categorical variables for current use of medication for cardiovascular disease, hypertension, lipidemia and diabetes (“no” or “yes”).

### Behavioural covariates

Smoking status was categorized on the basis of serum-cotinine levels from the physical examination. Participants completed two 24 hour diet recalls, which were coupled with US Department of Agriculture food composition data to determine mean daily total energy intake and mean saturated fat as a percentage of total energy intake. Mean daily alcohol intake was collapsed into sex-specific categories on the basis of US dietary guidelines. [16]

### Statistical analyses

In order to obtain population-representative findings, analyses were weighted using sample weights published for each NHANES cycle. Weighted descriptive characteristics were calculated on the full, fasting and OGTT samples (means, standard error, and frequency/percentages). Partial correlations were used to determine the correlations between the physical activity and sedentary variables and detect the presence of collinearity.

Numerous cardio-metabolic variables (all, except waist circumference and diastolic blood pressure) were positively skewed, so they were log-transformed prior to analyses. To aid interpretation, data were back-transformed from the log scale for presentation in the results. In addition, the physical activity variables (MVPA minutes and total physical activity) were log-transformed prior to analyses.

The partial correlations between sedentary and physical activity variables were adjusted for wear time, and showed that sedentary time had moderate to strong negative correlations with MVPA minutes and total physical activity (Spearman's rho = −0.52 (p<0.0001) and Spearman's rho = −0.78 (p<0.0001) respectively when controlled for wear time), which were below accepted thresholds for collinearity of 0.9 to 0.95 [17]. A series of linear regression models were progressively developed, in order to help understand the presence and magnitude of association between mean daily sedentary time and the cardio-metabolic variables. Each successive model incorporated new variables, whilst also retaining those from earlier models, to help reveal the importance of sedentary time as potential confounders were introduced to the models, i.e. Model 1 simply regressed sedentary time against each cardio-metabolic biomarker, adjusting only for accelerometer wear time; Model 2 additionally adjusted for socio-demographic variables; Model 3 additionally adjusted for medical history; Model 4 additionally adjusted for dietary and smoking behaviour; and Model 5 additionally adjusted for MVPA minutes. Model 6 was similar to Model 5, but instead of using MVPA minutes as an indicator of physical activity, it was adjusted for total physical activity based on total accelerometer physical activity counts (excluding sedentary counts). All models accounted for the weighting, stratification and clustering of the NHANES sample.

The specific covariates used for each biomarker were determined by running them in complex survey linear regression models. Sedentary minutes, accelerometer wear time, age, sex and ethnicity were used in all models, and the remaining socio-demographic variables were run in one model. Any socio-demographic variables which had a significant association with the particular biomarker being modelled (based on a significance level of *P*≤0.10) were retained and used in the models completed for this study. The same process was undertaken for medical history covariates in one model, and behavioural covariates in one model. The specific covariates retained for each biomarker are shown in the Table S1.

Potential interactions between sedentary time and age, sex and ethnicity and were examined for the biomarkers found to be significant associated with sedentary time in model 6.

In order to determine the relative strength of association between sedentary time and cardio-metabolic outcomes, the standardized β for sedentary time was compared with the standardized β for total physical activity.

To visually display the relationship between sedentary time and significantly associated cardio-metabolic outcomes, when corrected for MVPA minutes and total physical activity respectively, sedentary time was split into quartiles. Adjusted means and 95% confidence intervals (CI) were calculated using least squares means. An alpha of 0.05 was considered to indicate a statistically significant relationship between sedentary time and the cardio-metabolic biomarkers. Analyses were conducted using SAS version 9.2 (SAS Institute Inc., Cary, North Carolina, USA). Unless otherwise specified, an alpha value of *P*<0.05 was used to indicate significance.

## Results

A total of 4,618 NHANES participants met the inclusion criteria; their socio-demographic and behavioural characteristics are provided in Table 1.

A series of linear regression models were built, to determine the presence and magnitude of associations between mean daily sedentary time and each cardio-metabolic biomarker (Table 2). In the simplest model (Model 1 – in which the only covariate was accelerometer wear time), sedentary time was significantly associated with all 11 cardio-metabolic biomarkers. In all cases with the exception of diastolic blood pressure, higher sedentary time was associated with detrimental levels of the biomarkers. However, the relationship between sedentary time and each biomarker was very small, as indicated by the adjusted R^2^ values (0.003 to 0.038), showing that just 0.3 to 4% of the variability in cardio-metabolic biomarkers was explained by the model.

**Table 2:** Linear Regression Models Showing the Strength of the Association Between Total Daily Sedentary Time (in Hours) and Biomarkers in Adults in the 2003/04 and 2005/06 U.S. National Health and Nutrition Examination Survey.

Biomarker	Expected direction of relationship with sedentary time	Model 1. Sedentary hours, and wear time		Model 2. Sedentary hours, and wear time		Model 3. Sedentary hours, and wear time		Model 4. Sedentary hours, and wear time		Model 5. Sedentary hours, and wear time		Model 6. Sedentary hours, and wear time	
				+ sociodemographic^a^		+ sociodemographic^a^		+ sociodemographic^a^		+ sociodemographic^a^		+ sociodemographic^a^	
						+ medical history^b^		+ medical history^b^		+ medical history^b^		+ medical history^b^	
								+ behaviour^c^		+ behaviour^c^		+ behaviour^c^	
										+ MVPA minutes		+ Total activity counts, excluding sedentary	
		β for sed time (*P*)	Adjusted R^2^ for model	β for sed time (*P*)	Adjusted R^2^ for model	β for sed time (*P*)	Adjusted R^2^ for model	β for sed time (*P*)	β for sed time (*P*)	β for sed time (*P*)	Adjusted R^2^ for model	β for sed time (*P*)	Adjusted R^2^ for model
Waist circumference	+	1.45^***^	0.027	1.60^***^	0.165	1.27^***^	0.225	1.23^***^	0.250	0.53^**^	0.268	−0.37	0.264
LOG Systolic BP	+	0.013^***^	0.022	0.002	0.210	0.001	0.217	0.002	0.223	−0.001	0.225	−0.004	0.224
Diastolic BP	+	−0.40^**^	0.003	0.27	0.131	0.33*	0.136	0.33*	0.136	0.29	0.137	0.13	0.137
LOG HDL	−	−0.01^***^	0.003	−0.03^***^	0.192	−0.02^***^	0.205	−0.02^***^	0.250	−0.01^**^	0.258	0.003	0.258
LOG C-reactive protein	+	0.10^***^	0.021	0.11^***^	0.073	0.10^***^	0.087	0.10^***^	0.100	0.03*	0.121	−0.06*	0.119
LOG fasting Triglycerides	+	0.06^***^	0.033	0.07^***^	0.111	0.06^***^	0.123	0.06^***^	0.132	0.05^***^	0.136	0.04*	0.135
LOG fasting plasma glucose	+	0.01^***^	0.018	0.01^***^	0.139	0.005*	0.350	0.005*	0.357	0.001	0.360	−0.003	0.359
LOG Insulin	+	0.09^***^	0.037	0.12^***^	0.086	0.11^***^	0.137	0.11^***^	0.192	0.08^***^	0.208	0.02	0.208
LOG HOMA %B	+	0.04^***^	0.014	0.06^***^	0.072	0.06^***^	0.126	0.06^***^	0.166	0.05^***^	0.172	0.02	0.173
LOG HOMA %S	−	−0.09^***^	0.038	−0.12^***^	0.089	−0.11^***^	0.142	−0.11^***^	0.197	−0.08^***^	0.214	−0.02	0.214
LOG OGTT 2 h plasma glucose	+	0.04^**^	0.036	0.03^***^	0.144	0.03^**^	0.178	0.03^**^	0.201	0.02*	0.205	0.01	0.204

Abbreviations: β =  metric regression coefficient; BP =  blood pressure; HDL =  High-density lipoprotein; HOMA %B =  Homeostasis Model Assessment steady state beta cell function, HOMA %S =  Homeostasis Model Assessment insulin sensitivity, OGTT =  oral glucose tolerance test; sed time  =  sedentary time.

*P*<0.05; **P<0.01; ***P<0.001. *P* values are two-sided.

^a,b,c^ see Table S1 for a summary of the socio-demographic, medical history, smoking and dietary behaviour variables included in each model.

Models 2, 3 and 4 involved further covariates being added to the regression models (socio-demographic, plus medical history, plus dietary and smoking behaviour). Looking across these models in Table 2, a pattern for the R^2^ values to gradually increase is apparent, indicating that the more complex models better explained the variability in cardio-metabolic biomarkers. By Model 4, sedentary time was still significantly associated with 10 of the 11 cardio-metabolic biomarkers, although there was a clear pattern for the sedentary time metric regression coefficient to reduce in magnitude across the models, indicating that the relationship between sedentary time and cardio-metabolic biomarkers weakened as confounders were accounted for.

Model 5 was similar to the linear regression model previously reported in the literature, [11] where the relationship between sedentary time and cardio-metabolic biomarkers was adjusted for duration of MVPA behaviour, in addition to socio-demographic, medical history, and dietary and smoking behaviour variables. In this model, the sedentary time metric regression coefficient further reduced in magnitude, so that sedentary time was significantly associated with 8 of the 11 cardio-metabolic biomarkers. In all cases in which a significant association was present, higher sedentary time was associated with detrimental differences in the cardio-metabolic biomarkers.

Finally, Model 6 examined the relationship between sedentary time and cardio-metabolic biomarkers, adjusting for total physical activity (total daily accelerometer counts, excluding sedentary counts), in addition to socio-demographic, medical history, and dietary and smoking behaviour variables. In this model, sedentary time is significantly associated with 2 of the 11 cardio-metabolic biomarkers: C-reactive protein and triglycerides. In both cases, the magnitude of the relationship is very small, as indicated by metric regression coefficient values of −0.06 and 0.04, respectively. Interestingly, in the case of C-reactive protein, the direction of the relationship was reversed, so that higher sedentary time was associated with *favourable* differences in C-reactive protein. There were no significant interactions between sedentary time and sex, ethnicity or age for either triglycerides or C-reactive protein.

In order to determine the relative magnitude of the association between the cardio-metabolic outcomes and sedentary time and total physical activity, the standardized β for sedentary time was compared with the standardized β for total physical activity (Table 3). Total physical activity was most strongly and consistently associated with the biomarkers (significant associations in 9 out of 11 biomarkers, absolute standardized β = 0.04–0.30). In all cases, higher physical activity was associated with favourable levels of the biomarkers. There were, however, few and weak associations between sedentary time and biomarkers (significant association in 2 out of 11 biomarkers, absolute standardized β = 0.02–0.12). As previously noted, in one of these two cases (C-reactive protein), higher sedentary time was associated with favourable differences in the biomarker.

**Table 3:** The Relative Strength of Association Between Sedentary Time, Total Physical Activity and Cardio-Metabolic Biomarkers in Adults in the 2003/04 and 2005/06 U.S. National Health and Nutrition Examination Survey.

Biomarker	Sedentary time Standardised β	Total physical activity Standardised β
Waist circumference	−0.05	−0.26^***^
LOG Systolic BP	−0.05	−0.08^**^
Diastolic BP	0.02	−0.04
LOG HDL	0.02	0.19^***^
LOG C-reactive protein	−0.09^**^	−0.30^***^
LOG fasting Triglycerides	0.12^**^	−0.12^**^
LOG fasting plasma glucose	−0.03	−0.11^**^
LOG Insulin	0.02	−0.27^***^
LOG HOMA %B	0.07	−0.18^***^
LOG HOMA %S	−0.04	0.27^***^
LOG OGTT 2 h plasma glucose	0.05	−0.12

Abbreviations: BP =  blood pressure; HDL =  High-density lipoprotein; HOMA %B =  Homeostasis Model Assessment steady state beta cell function, HOMA %S =  Homeostasis Model Assessment insulin sensitivity, OGTT =  oral glucose tolerance test; NIM =  not included in model; CVD =  cardiovascular disease.

*P*<0.05; **P<0.01; ***P<0.001. *P* values are two-sided.

Models were adjusted for socio-demographic, medical history and smoking, alcohol and dietary behaviour. Please see Table S1 for full list of covariates included in the model for each cardio-metabolic biomarker.

**Figure 1:**
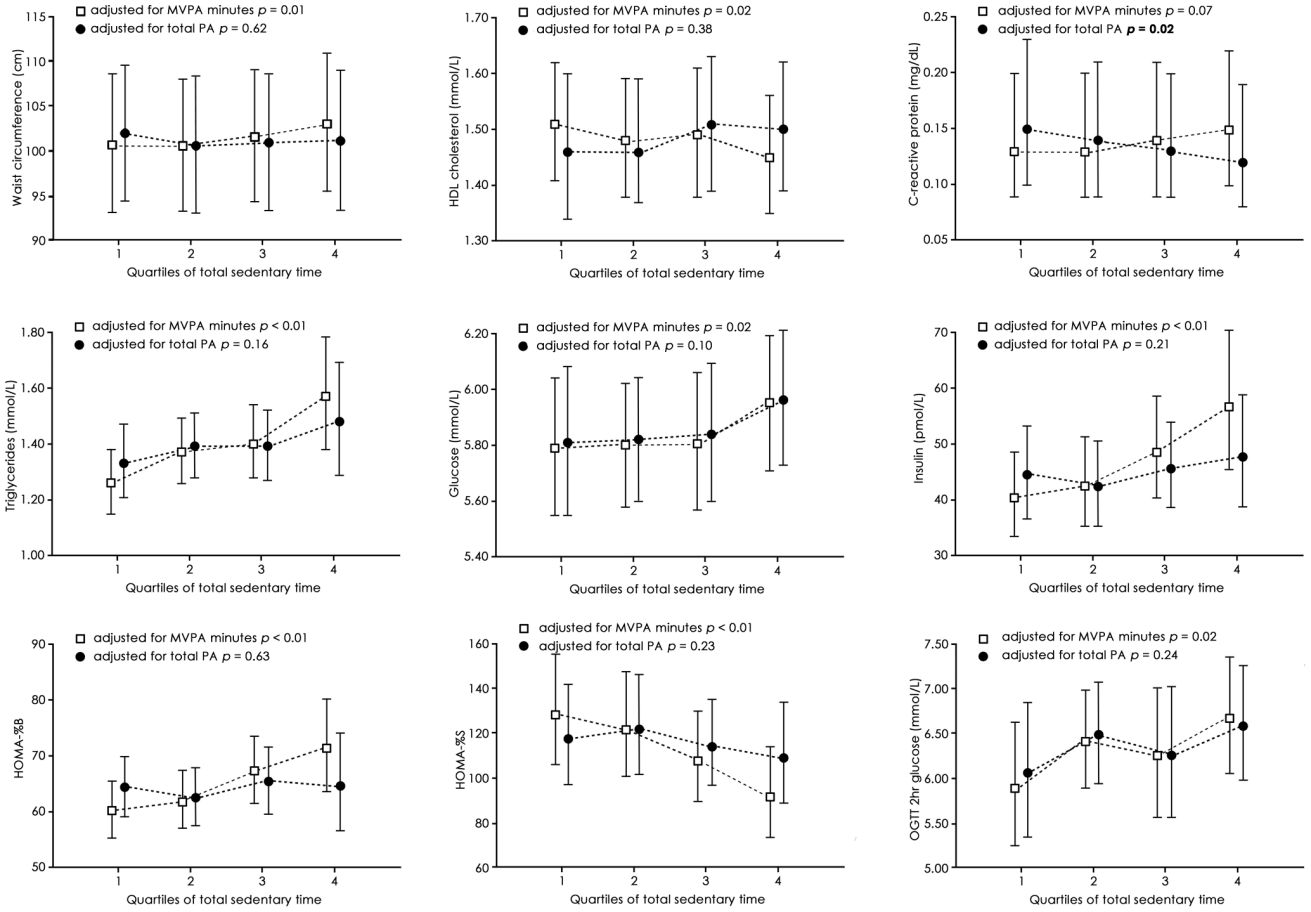
Associations between total sedentary time and cardio-metabolic biomarkers, corrected for two different measures of physical activity (MVPA minutes, and total physical activity) in adults in the 2003/04 and 2005/06 U.S. National Health and Nutrition Examination Survey. Only biomarkers where significant associations were observed are shown. MVPA =  moderate to vigorous physical activity; HDL =  High-density lipoprotein; HOMA %B =  Homeostasis Model Assessment steady state beta cell function, HOMA %S =  Homeostasis Model Assessment insulin sensitivity, OGTT =  oral glucose tolerance test.

In an effort to understand the clinical significance of the relationships between sedentary time and cardio-metabolic biomarkers, and how these apparent relationships differed depending on whether analyses were corrected for MVPA minutes or total physical activity, we undertook one further analysis. We divided sedentary time into quartiles, as has been done by previous investigators, [11] and calculated the adjusted means (95% CI) for the biomarkers, using the same covariates used in Table 2 Model 5 (MVPA minutes) and Model 6 (total physical activity). Sedentary time quartile cutpoints were 6.88, 8.14 and 9.43 hours/day. As can be seen from Figure 1, when analyses were corrected for MVPA minutes, 8 out of 11 biomarkers showed significant detrimental relationships with sedentary time (all biomarkers except systolic blood pressure, diastolic blood pressure and C-reactive protein). However, when analyses were corrected for total physical activity, the associations were no longer statistically significant for any of these 8 biomarkers. Once total activity counts were accounted for, only one biomarker had a significant relationship with sedentary time, C-reactive protein, and this biomarker showed a *favourable* relationship, that is, as sedentary time increased, C-reactive protein significantly decreased (*P* = 0.02).

## Discussion

This study showed that while there are weak relationships between cardio-metabolic biomarkers and sedentary behaviour when analyses are adjusted for MVPA minutes, the associations effectively disappear when analyses are adjusted for total physical activity. The exceptions to this are C-reactive protein, which show a minute, favourable association with sedentary time, and triglycerides, which show a minute, detrimental association with sedentary time.

While previous studies have found statistically significant relationships between sedentary behaviour and biomarkers of cardio-metabolic risk, [11], [18], [19] few have drawn attention to the fact that these relationships are very weak, and may not be of major clinical significance. It is possible that the relationships between sedentary behaviour and biomarkers are due to residual confounding. Accelerometry-based measures of physical activity (such as MVPA minutes and total physical activity counts) are quite crude. As such, residual confounding may account for associations between sedentary time and health parameters even after adjustment for physical activity.

Alternatively, it is possible that they are true associations, albeit of very small magnitude. We produced Figure 1 to aid interpretability of the relationships between sedentary behaviour and health biomarkers. Looking at triglycerides (the only biomarker that maintained a detrimental association with sedentary time once corrected for total physical activity in Table 2), there was a very small difference between the triglyceride levels in the most sedentary versus the least sedentary segments of the population – just 0.15 mmol per liter variation occurred across the sedentary behaviour spectrum. It is difficult to argue that such a difference is of clinical significance considering that a change of 0.28 mmol/L is required to shift someone from one risk band to another (e.g. from “high” to “borderline”, or from “borderline” to “normal”; based on the definitions of <1.69 mmol/L =  normal, 1.69−2.25 =  borderline, >2.25 mmol/L =  high. [20]). Furthermore, the small difference in triglycerides association with quite marked variation in sedentary behaviour (4.5 h difference in sedentary time between the median values for the least sedentary and most sedentary quartiles) suggests the relationship is of minimal population health significance.

The study has two main implications. Firstly, further research is needed in other cross-sectional datasets, and using other research designs, to replicate our findings. Appropriately designed experimental studies will help our understanding of potential mechanisms underpinning the link between sedentary behaviour and health risk. Such studies could definitively answer the question of whether overall activity level is the underlying factor, or alternatively, whether physical activity and sedentary behaviour are indeed two independent entities. In recent years public health messages have increasingly encouraged people to reduce sitting behaviour (and often, replacing it with stationary standing); our findings suggest that a better message would be to encourage movement at a low intensity. Secondly, our study has important methodological implications for future research examining the health effects of physical activity and sedentary behaviour. Focussing on MVPA ignores a large part of the intensity spectrum of physical activity, and a part which can have a significant impact on health. Failure to account for the full spectrum of physical activity may inadvertently attribute health effects to sedentary behaviour which may in fact be due to lower total physical activity.

A strength of the current study was the use of a large, nationally representative dataset. Physical activity and sedentary behaviour were measured objectively using accelerometers, which minimized the bias associated with recall. [21] Accelerometer data handling ensured that sedentary time and physical activity were estimated with greatest accuracy possible. In particular, the censoring of consecutive minutes of zero counts allowed the proper estimation on non-wear time; these minutes were removed from total activity, thus preventing the overestimation of time spent in sedentary.

Furthermore, the comprehensive nature of the NHANES dataset allowed us to statistically control for numerous covariates, including ethnicity, socioeconomic status, medical history and diet (including saturated fat and alcohol intake), which if not included, may have confounded the results. Limitations of the study should also be acknowledged. This study is cross-sectional, therefore only associations, and not causation, can be determined.

Some may argue that a correlation of Spearman's rho = −0.78 between sedentary minutes and total activity counts less sedentary represents collinearity, threatening the validity of findings. Collinearity diagnostic tests were undertaken to explore this issue. The Variance Inflation Factor (VIF) for total physical activity and sedentary time in our models was 5.2, which is well below the threshold of VIF >10 generally accepted to indicate collinearity [22], [23], [24]. Furthermore, we examined the stability of the regression coefficients by running statistical models using sedentary time and total physical activity separately, and together. Should collinearity have been present, we would have expected the regression estimates to change erratically in the combined model compared with the separate models. However, the model outputs were remarkably consistent across the separate and combined models, indicating that the analyses presented within this paper are robust. The results of these analyses are presented in the Table S2.

In conclusion, while there are significant (albeit weak) relationships between sedentary behaviour and cardio-metabolic risk factors with or without adjustment for MVPA minutes, these relationships dissipate when adjusted for overall physical activity. Thus it appears that sedentary behaviour may not have health effects independent of physical activity.

## Supporting Information

Table S1 Socio-Demographic, Medical History and Behavioral Covariates Used in Linear Regression Models With Sedentary Minutes, Accelerometer Wear Time, Age, Sex and Ethnicity to Determine Which Covariates to Retain. Analyses were undertaken for the US adult population in the 2003/04 and 2005/06 U.S. National Health and Nutrition Examination Survey. Retained if *P*<0.10 (two-sided).
(DOCX)

Table S2 Regression Coefficients for Sedentary Time and Total Physical Activity, in Regression Models Undertaken for Sedentary Time Alone, Total Physical Activity Alone, and Sedentary and Total Physical Activity Together.
(DOCX)
