# Supplementary material for: Reconsidering the Sedentary Behaviour Paradigm
Source: PLoS One. 2014 Jan 15;9(1):e86403. doi: 10.1371/journal.pone.0086403 (PMC3893290; doi:10.1371/journal.pone.0086403)
Supplement: Table S1 — Socio-Demographic, Medical History and Behavioral Covariates Used in Linear Regression Models With Sedentary Minutes, Accelerometer Wear Time, Age, Sex and Ethnicity to Determine Which Covariates to Retain. Analyses were undertaken for the US adult population in the 2003/04 and 2005/06 U.S. National Health and Nutrition Examination Survey. Retained if P<0.10 (two-sided). (DOCX) [file pone.0086403.s001.docx]

**Table S1** Socio-Demographic, Medical History and Behavioral Covariates Used in Linear Regression Models With Sedentary Minutes, Accelerometer Wear Time, Age, Sex and Ethnicity to Determine Which Covariates to Retain. Analyses were undertaken for the US adult population in the 2003/04 and 2005/06 U.S. National Health and Nutrition Examination Survey. Retained if *P* < 0.10 (two-sided).

|  | **Socio-demographic** | **Medical history** | **Behavior** |
| --- | --- | --- | --- |
| Waist circumference | age-squared, educational attainment | relative with diabetes, on CVD meds, diabetes meds, ever been told cancer, ever been told diabetes, ever been told CVD | smoking status, saturated fat as % of total, alcohol intake |
| LOG Systolic BP | educational attainment | relative with diabetes, on CVD meds, HT meds, ever been told diabetes | smoking status, total energy intake, alcohol intake |
| Diastolic BP | age-squared, educational attainment | diabetes meds, ever been told CVD |  |
| LOG HDL | age-squared, poverty-income ratio, educational attainment | relative with diabetes, on CVD meds, diabetes meds, ever been told diabetes | smoking status, alcohol intake |
| LOG C-reactive protein | age-squared, poverty-income ratio, educational attainment | relative with diabetes, on CVD meds, lipidemic meds, ever been told diabetes | smoking status, total energy intake, saturated fat as % of total, alcohol intake |
| LOG fasting Triglycerides | age-squared, educational attainment | relative with diabetes | smoking status, total energy intake, alcohol intake |
| LOG fasting plasma glucose | age-squared, educational attainment | relative with diabetes, on CVD meds, lipidemic meds, ever been told diabetes, ever been told CVD | smoking status, saturated fat as % of total, alcohol intake |
| LOG Insulin | age-squared, educational attainment | relative with diabetes, on CVD meds, lipidemic meds, ever been told cancer | smoking status, total energy intake, saturated fat as % of total, alcohol intake |
| LOG HOMA %B | educational attainment | relative with diabetes, on CVD meds, lipidemic meds, ever been told diabetes, ever been told CVD | smoking status, total energy intake, saturated fat as % of total, alcohol intake |
| LOG HOMA %S | age-squared, educational attainment | relative with diabetes, on CVD meds, lipidemic meds, ever been told cancer | smoking status, total energy intake, saturated fat as % of total, alcohol intake |
| LOG OGTT 2 h plasma glucose | educational attainment | relative with diabetes, on CVD meds, lipidemic meds | smoking status, saturated fat as % of total |

Abbreviations: BP = blood pressure; HDL = High-density lipoprotein; HOMA %B = Homeostasis Model Assessment steady state beta cell function; HOMA %S = Homeostasis Model Assessment insulin sensitivity; OGTT = oral glucose tolerance test; CVD = cardiovascular disease; HT = hypertension.
