# Supplementary material for: Reconsidering the Sedentary Behaviour Paradigm
Source: PLoS One. 2014 Jan 15;9(1):e86403. doi: 10.1371/journal.pone.0086403 (PMC3893290; doi:10.1371/journal.pone.0086403)
Supplement: Table S2 — Regression Coefficients for Sedentary Time and Total Physical Activity, in Regression Models Undertaken for Sedentary Time Alone, Total Physical Activity Alone, and Sedentary and Total Physical Activity Together. (DOCX) [file pone.0086403.s002.docx]

**Table S2** Regression Coefficients for Sedentary Time and Total Physical Activity, in Regression Models Undertaken for Sedentary Time Alone, Total Physical Activity Alone, and Sedentary and Total Physical Activity Together.

|  | **Sedentary Only** |  |  | **Total Physical Activity only** |  |  | **Sedentary and Total Physical activity** |  |  |  |  |
| --- | --- | --- | --- | --- | --- | --- | --- | --- | --- | --- | --- |
|  | Adjusted R^2^ | β for Sed time (p) | SE for Sed time | Adjusted R^2^ | β for TPA (p) | SE for TPA | Adjusted R^2^ | β for Sed time (p) | SE for Sed time | β for TPA (p) | SE for TPA |
| Waist circumference | 0.25 | 1.23 (<0.0001) | 0.1378 | 0.264 | -6.08 (<0.0001) | 0.3646 | 0.264 | -0.37 (0.1755) | 0.2673 | -7.11 (<0.0001) | 0.8098 |
| LOG Systolic BP | 0.223 | 0.002 (0.2819) | 0.0014 | 0.224 | -0.01 (0.0364) | 0.0056 | 0.224 | -0.004 (0.1469) | 0.0024 | -0.02 (0.0258) | 0.0095 |
| Diastolic BP | 0.136 | 0.33 (0.0354) | 0.1516 | 0.137 | -1.26 (0.0472) | 0.6087 | 0.137 | 0.13 (0.5819) | 0.2271 | -0.91 (0.3387) | 0.9341 |
| LOG HDL | 0.25 | -0.02 (<0.0001) | 0.0026 | 0.258 | 0.09 (<0.0001) | 0.0092 | 0.258 | 0.003 (0.6114) | 0.0055 | 0.10 (<0.0001) | 0.0197 |
| LOG C-reactive protein | 0.1 | 0.10 (<0.0001) | 0.0136 | 0.118 | -0.52 (<0.0001) | 0.0498 | 0.119 | -0.06 (0.0165) | 0.0236 | -0.69 (<0.0001) | 0.0847 |
| LOG fasting Triglycerides | 0.132 | 0.06 (<0.0001) | 0.0086 | 0.132 | -0.23 (<0.0001) | 0.0346 | 0.135 | 0.04 (0.0129) | 0.0138 | -0.13 (0.0245) | 0.0544 |
| LOG fasting plasma glucose | 0.357 | 0.005 (0.0189) | 0.0019 | 0.359 | -0.03 (0.0011) | 0.0073 | 0.359 | -0.003 (0.3118) | 0.0029 | -0.03 (0.0047) | 0.0114 |
| LOG Insulin | 0.192 | 0.11 (<0.0001) | 0.0119 | 0.207 | -0.47 (<0.0001) | 0.0474 | 0.208 | 0.02 (0.2057) | 0.015 | -0.42 (<0.0001) | 0.0589 |
| LOG HOMA %B | 0.166 | 0.06 (<0.0001) | 0.0078 | 0.172 | -0.25 (<0.0001) | 0.0299 | 0.173 | 0.02 (0.0895) | 0.0125 | -0.19 (0.0003) | 0.0463 |
| LOG HOMA %S | 0.197 | -0.11 (<0.0001) | 0.0118 | 0.214 | 0.48 (<0.0001) | 0.0473 | 0.214 | -0.02 (0.2678) | 0.0147 | 0.43 (<0.0001) | 0.0584 |
| LOG OGTT 2 h plasma glucose | 0.201 | 0.03 (0.0033) | 0.0083 | 0.204 | -0.11 (0.0028) | 0.031 | 0.204 | 0.01 (0.491) | 0.0151 | -0.08 (0.1689) | 0.0554 |

Abbreviations: BP = blood pressure; HDL = High-density lipoprotein; HOMA %B = Homeostasis Model Assessment steady state beta cell function, HOMA %S = Homeostasis Model Assessment insulin sensitivity, OGTT = oral glucose tolerance test; NIM = not included in model; CVD = cardiovascular disease.

Models were adjusted for socio-demographic, medical history and smoking, alcohol and dietary behaviour. Please see Supplementary File Table 1 for full list of covariates included in the model for each cardio-metabolic biomarker.
